# Management of Atlantoaxial Instability Due to a Pathological Fracture of the Axis

**DOI:** 10.7759/cureus.8951

**Published:** 2020-07-01

**Authors:** Vineeth Thirunavu, Nader S Dahdaleh

**Affiliations:** 1 Department of Neurological Surgery, Feinberg School of Medicine, Northwestern University, Chicago, USA; 2 Department of Neurological Surgery, Feinberg School of Medicine Northwestern University, Chicago, USA

**Keywords:** metastatic breast cancer, spinal metastasis, axis fracture, c2 fracture, crown halo vest, occipitocervical fusion, craniovertebral junction

## Abstract

Metastases to the upper cervical spine are uncommon, and subsequent instability is rare. We report the details of a patient with metastatic breast cancer to the axis manifesting as a pathologic fracture and C1/C2 instability. This was treated by preoperative reduction to appropriate alignment with use of crown halo traction, followed by posterior occipitocervical fusion and postoperative radiation therapy.

## Introduction

The greater blood supply and volume of bone in the thoracic and lumbar spine makes metastases to the cervical spine, including the upper cervical spine, an uncommon occurrence [1]. Only about 0.5% of metastatic spinal lesions are to the cervical spine [2]. Management modalities for spinal metastases depend on multiple factors, including prognosis, functionality, type of primary tumor, disease control, and spinal biomechanical stability [3]. One management option is radiation therapy, which is offered without or subsequent to surgical intervention.

The decision to offer a patient surgical intervention primarily depends on his or her prognosis and functional status and secondarily on the presence of a metastatic lesion causing a neurological deficit and/or biomechanical instability [3,4]. The spinal instability neoplastic scoring system was developed to assist the treating physician in determining the necessity of surgical intervention and stabilization, taking into consideration the type of tumor, presence of neural element compression, and presence of biomechanical instability [4].

The upper cervical spine, which includes the occipital condyles, atlas, and axis, is unique in regards to its bony anatomy, relationship of neurovascular structures, and biomechanics [5]. Since this region is the most mobile in the spine, accounting for 50% of neck motion, it is more prone to instability when it is involved with a metastasis, especially those causing a pathological fracture. Moreover, pathological fractures of the axis, in particular, are at high risk for causing instability, significant pain, and morbidity [1,6,7].

When surgical stabilization of the upper cervical spine, namely the axis, is deemed appropriate, there are different surgical approaches that can be offered. These include posterior and anterior instrumentation, occipitocervical fixation, C1 to subaxial cervical fixation, vertebroplasty, transpedicular corpectomy, a high cervical lateral approach between the sternocleidomastoid and internal jugular vein, and transmandibular/transoral approach, each with their own benefits and risks [1,6,8-12].

In this report, we describe a patient who suffered a pathological fracture of the axis, atlantoaxial instability, and upper cervical spinal deformity as a result of breast cancer metastases. We detail the surgical treatment with occipitocervical fusion and emphasize the use of preoperative crown halo traction for reduction of the subluxation in order to achieve appropriate alignment of the upper cervical spine.

## Case presentation

**Figure 1:**
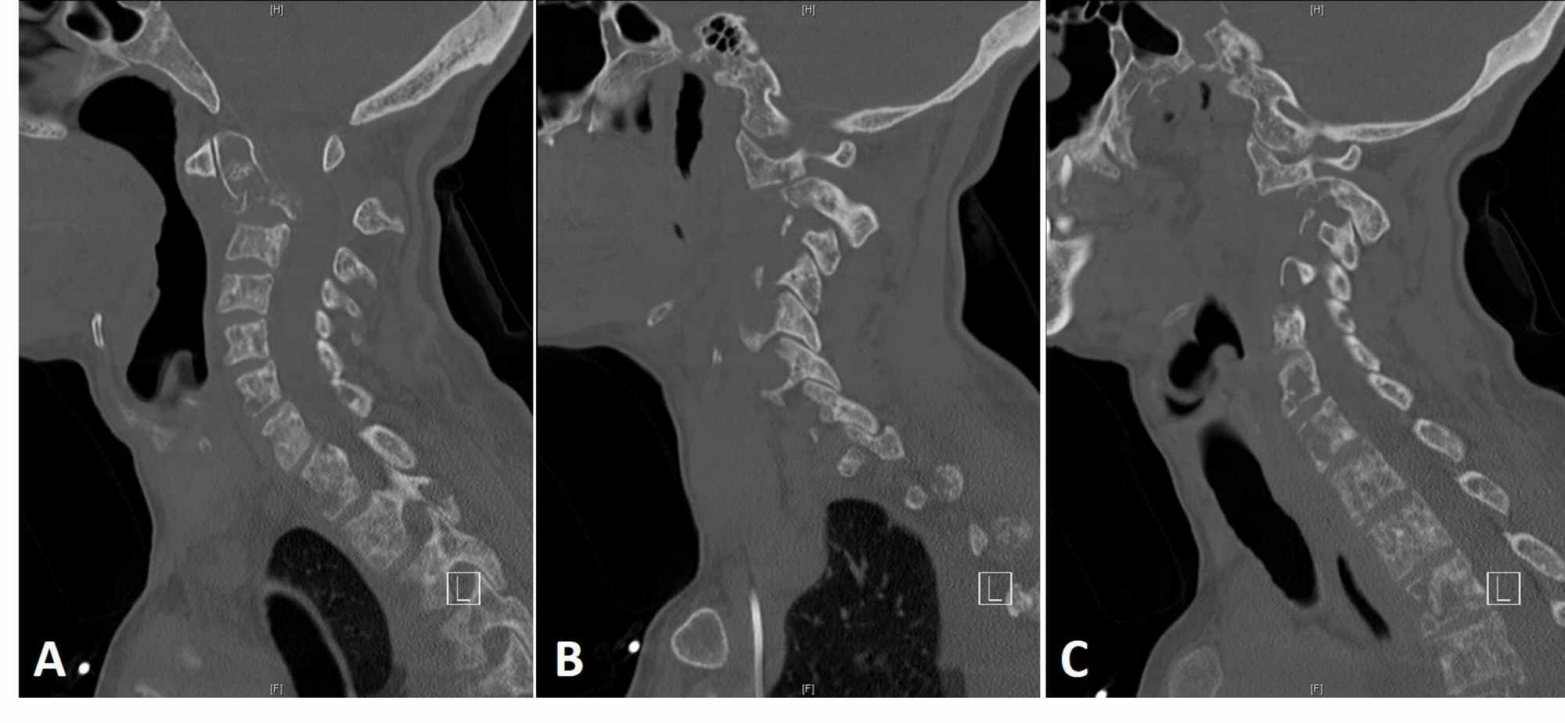
Preoperative CT of the cervical spine without contrast CT of the cervical spine without contrast showing acute, displaced, and impacted pathologic fracture of the base of the odontoid process extending into the C2 body (A mid-sagittal), anterior subluxation of the lateral mass of C1 on C2, and abnormal widening of the interval between the posterior arch of C1 and C2 spinous process (B left and C right parasagittal).

**Figure 2:**
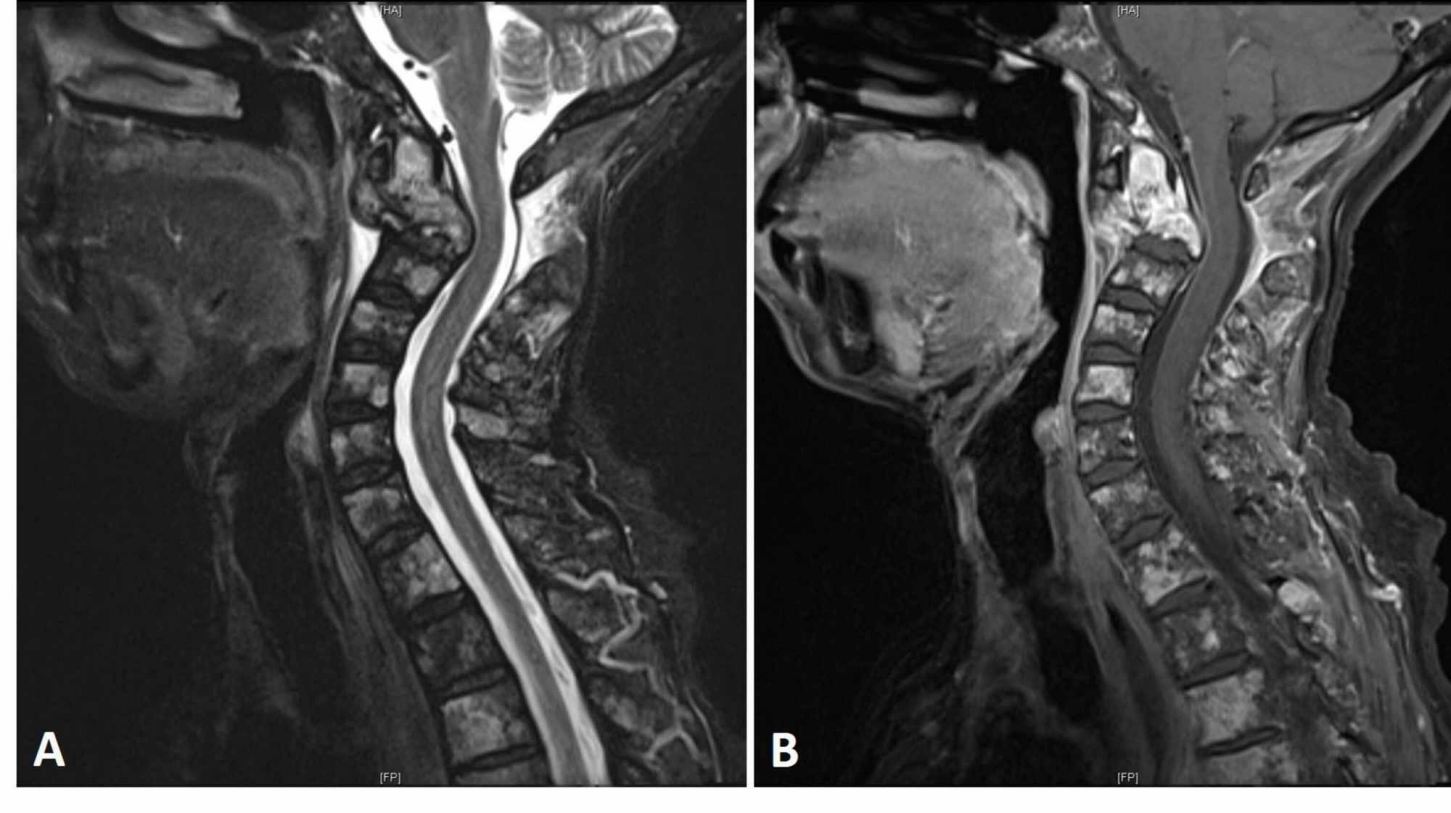
Preoperative MRI of the cervical spine with contrast MRI of the cervical spine with contrast showing extensive osseous metastatic disease and an upper cervical spinal kyphotic deformity due to the C2 pathological fracture (A: sagittal T2, and B: sagittal T1 postcontrast).

A 53-year-old woman with a known history of metastatic breast cancer managed with docetaxel/bevacizumab and Zometa presented with three weeks of upper cervical pain. She also had complaints of right-sided numbness to her face and neck and decreased range of motion. Her upper neck pain was so severe that she could not sit up for more than one minute before she needed to lay down for pain relief. Due to the severe pain, she was admitted to the hospital for workup and management. Her neurological examination was normal. CT and an MRI of the cervical spine revealed diffuse osseous metastatic disease of the cervical spine as well as a pathological fracture of axis with collapse and retropulsion causing significant atlantoaxial subluxation and deformity at the craniovertebral junction. There was also evidence for bony disease involving the skull base at the level of the foramen magnum as well as the subaxial spine (Figures 1, 2).

Case management

A multidisciplinary meeting that involved medical and radiation oncology and neurological surgery was conducted. The patient’s estimated survival was at least one year, and her Karnofsky score was 100. Given that the pathological fracture was causing atlantoaxial instability, the treatment algorithm included surgical stabilization.

Use of Crown Halo Traction

**Figure 3:**
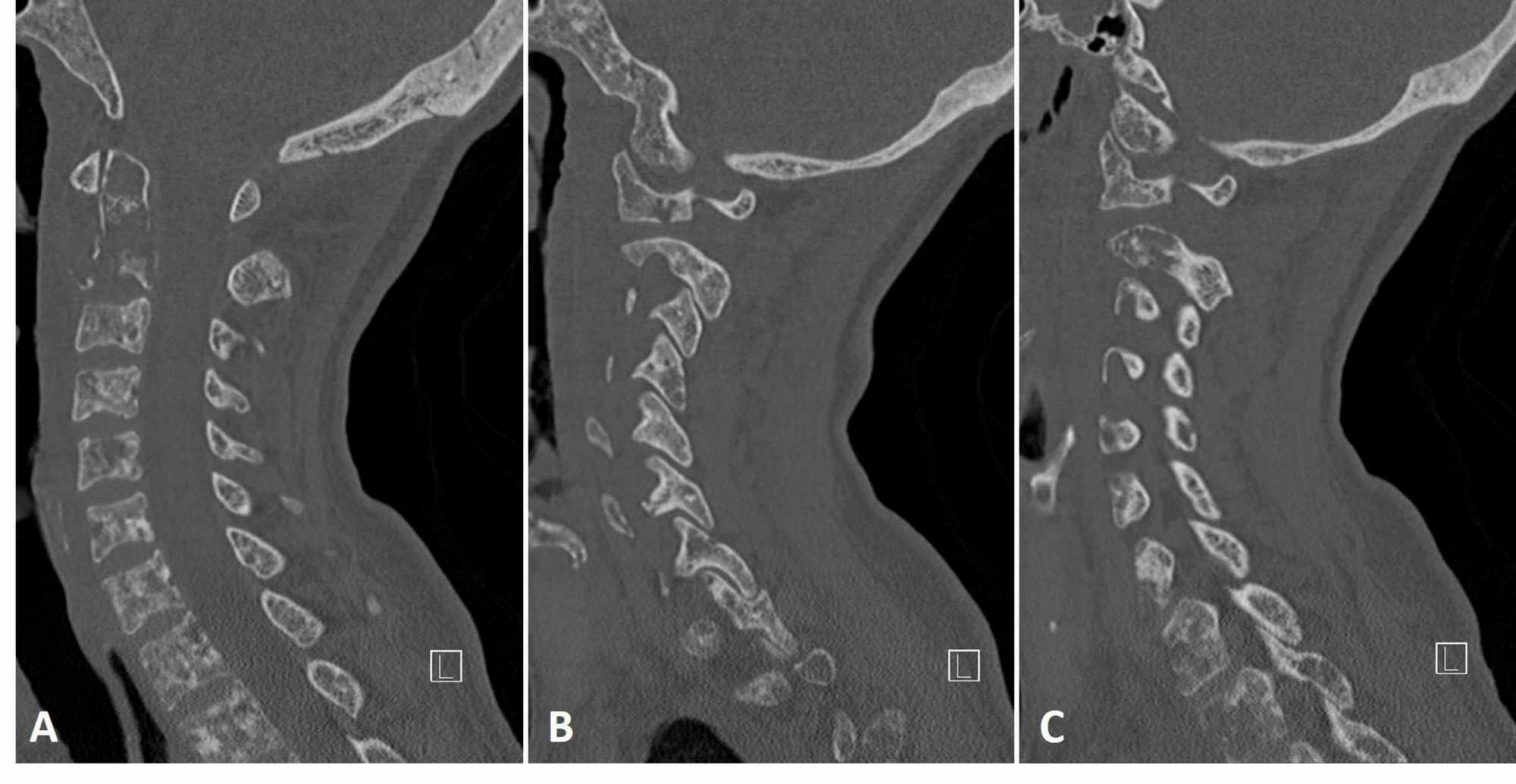
CT postapplication of crown halo traction CT postapplication of crown halo traction and subsequent vest placement showing realignment of the upper cervical spine (A: mid-sagittal) and reduction and realignment of the atlantoaxial joints bilaterally (B: left parasagittal and C: right parasagittal).

Due to evidence of instability of the craniovertebral junction requiring surgical stabilization, the patient was initially transferred to the neurosurgical ICU for crown halo traction to reduce the chronic subluxation. After being placed in a 24-hour crown halo traction of 15 pounds, both her upper cervical pain and C1/C2 alignment on lateral x-ray improved. With a successful reduction of C1-C2 subluxation, the crown halo traction was converted into a crown halo vest. The patient was locked into the crown halo vest for a day before operative stabilization was pursued. Subsequent CT of the cervical spine showed an appropriate reduction. The patient’s neck pain improved, and her swallowing was formally assessed as normal (Figure 3).

Operative Details

**Figure 4:**
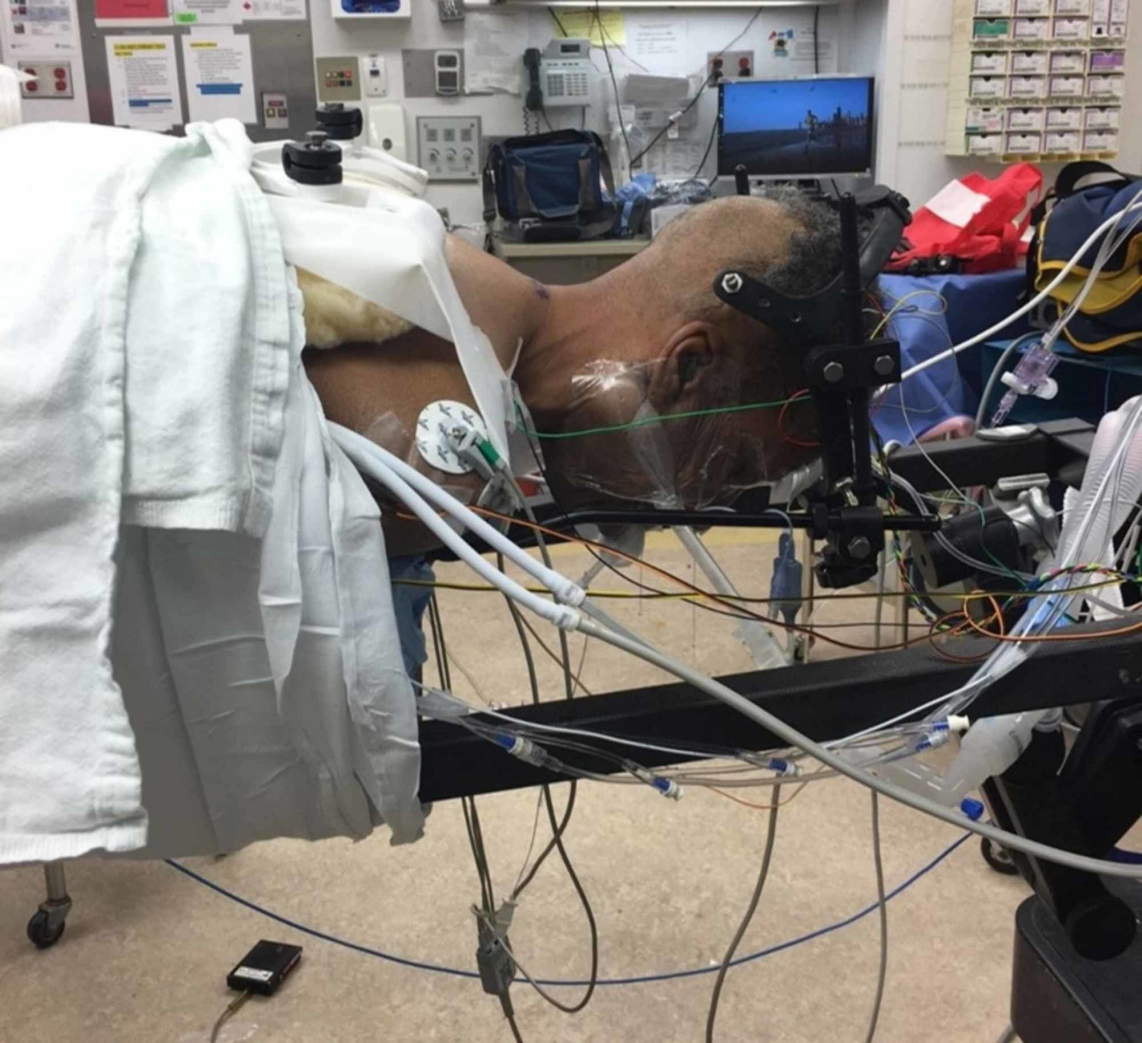
Intra-operative image of positioning and setup Intra-operative image illustrating prone positioning with the use of crown halo attached to a Mayfield (Integra LifeSciences Corporation, Plainsboro, NJ) holder via an OSI adapter on an open Jackson table.

The patient was subsequently taken to the operating room for posterior occipitocervical fusion to stabilize her upper neck. The patient was placed in the prone position on the open OSI Jackson table (Mizuho OSI, Union City, CA) while in her crown halo vest, and the crown halo was connected to the OSI table with an appropriate OSI adapter. Both arms and shoulders were tucked (Figure 4). An incision line was then made from the inion to the spinous processes of C6, and the posterior arch of C1 and the C1-C2 joint were exposed carefully by staying in the midline. The occipital plate was then placed with three screws along the midline keel with a transverse bar on either side to augment and maximize the points of fixation. Bilateral C2 pars intra-articular screws were then placed. Following that, bilateral C3 and C5 lateral mass screws were placed. No hardware was placed at C4 due to extensive bone involvement with metastatic disease. Two hinged rods were then used to connect the occipital plate with the lateral mass screw heads on both sides. Posterolateral fusion was then completed by applying a mixture of corticocancellous bone allograft and demineralized bone matrix that was laid above decorticated bone at the level of the occiput, posterior arch of the atlas, and facets of C3-C5. The wound was then closed in layers. The patient was then placed in the supine position and taken off the crown halo vest and placed in a rigid Miami J collar (Ossur hf, Reykjavík, Iceland). She was extubated successfully. The estimated blood loss was 100 mL.

Postoperative Details

The patient recovered well and was transitioned from intravenous pain medications to oral Tramadol. The patient was discharged with home physical therapy after clearance from physical and occupational therapy.

**Figure 5:**
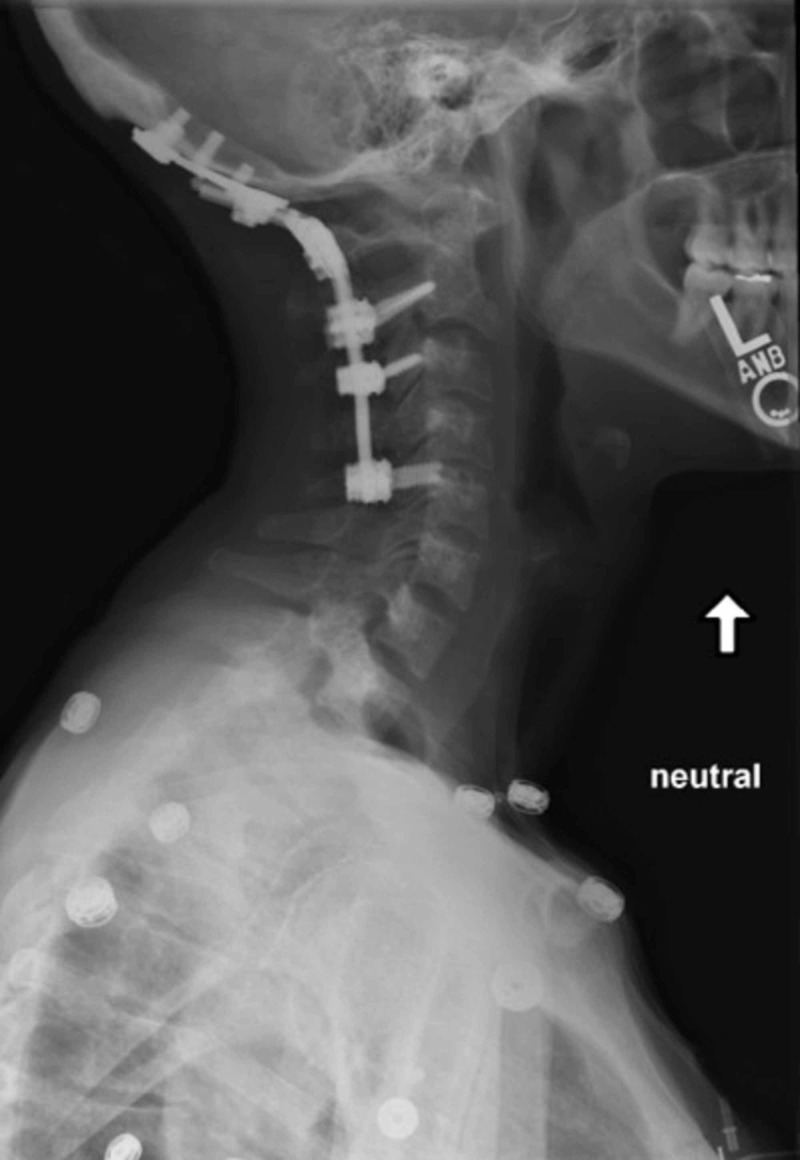
Postoperative lateral X-ray of the cervical spine Postoperative lateral X-ray of the cervical spine obtained at one year showing posterior occipitocervial fusion from the occiput to C5 with intact hardware and restored alignment.

**Figure 6:**
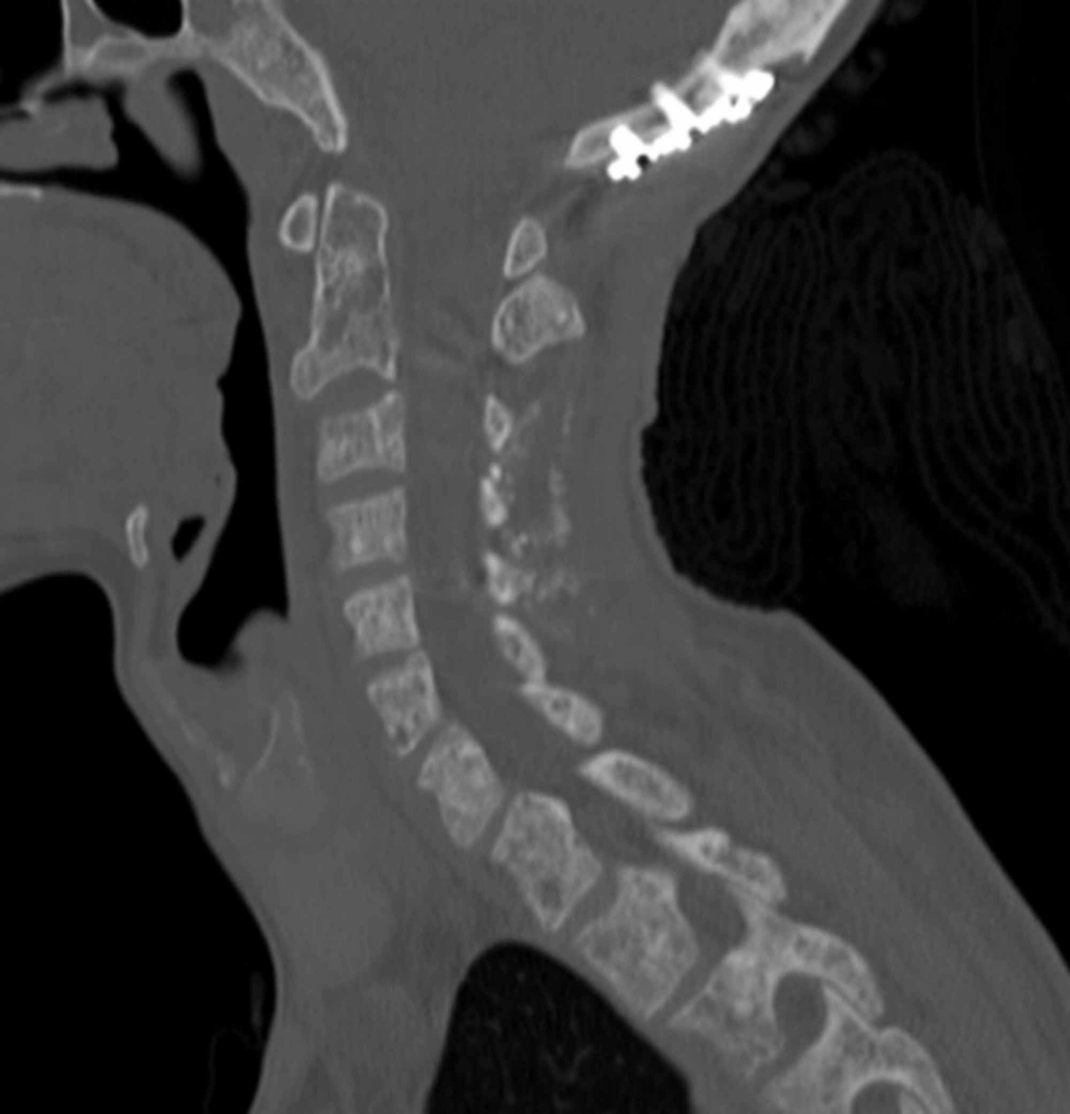
Postoperative CT of the cervical spine at one-year follow-up Follow-up CT of the cervical spine at one year showing posterior spinal fusion hardware from occiput to C5 showing appropriate alignment and reconstitution of bony integrity in the upper and subaxial cervical spine.

She returned to the radiation oncology center six weeks postop to receive palliative radiation therapy of 30 Gy in 3 Gy daily fractions over 10 days. The treatment was tolerated well and completed without any complications. At her one-year follow-up appointment, her neck pain was minimal. An upright cervical spine X-ray showed posterior fusion from occiput to C5 with intact hardware and normal alignment (Figure 5). CT showed improved alignment of the pathological fracture of the C2 vertebral body and decreased widening between the posterior arch of C1 and C2 spinous processes. There was also reconstitution of bony integrity at the upper and subaxial cervical spine (Figure 6). Unfortunately, the patient passed away 20 months following surgery due to hypoxic respiratory failure secondary to metastatic breast cancer.

## Discussion

Metastases to the upper cervical spine are rare but can often cause biomechanical instability when they occur [2,3]. If the spine is rendered unstable as a result of a pathological fracture, then surgical stabilization is warranted in order to preserve neurological function and improve pain [8]. In this case, there was not only biomechanical instability but also a form of deformity of the upper cervical spine created by the fracture. This required preoperative reduction using crown halo traction. Cervical braces and conservative management may be used in some cases of upper cervical spinal fractures. However, pain relief is often not adequate and complications may occur [13]. Studies have found cervical collar complication rates ranging from 0% to 44.4% and higher 30-day mortality rates for conservative management compared to operative intervention [14,15]. Additionally, the prolonged usage of crown halo vest is also associated with high complication rates in the elderly, with a notable complication being dysphagia [16,17]. In our case, the short-term usage of a crown halo vest allowed for stabilization of the patient’s neurologic status and facilitated the treatment of a potentially life-threatening injury. The acute placement of crown halo vest has been shown to provide immediate cervical stabilization and to aid diagnostic workup and preparation for surgery in patients similar to the one in our case [18].

After the placement of crown halo vest, usage of posterior occipitocervical fusion was indicated because it allows for robust stabilization and sound biomechanical construct for patients that can often last the lifetime of a patient with low risk of reoperation [8]. In the case of our patient, we chose to include the occiput as our cranial fixation point rather than the atlas. This was because the atlas was involved with metastases, and we were not confident that atlantal lateral mass screws would provide adequate fixation to combat the instability and excessive motion at the atlantoaxial joint. Following the operation, the patient’s neck pain improved significantly, and she subsequently underwent radiation treatment alongside optimization of her bone health. In a study that reported on five patients with metastases to the axis (three metastatic breast, one multiple myeloma, and one unknown carcinoma), the usage of posterior instrumentation in addition to a variety of stabilization options, including occipitocervical fixation, resulted in pain relief and adequate palliation for all patients [1]. Similarly, our patient maintained a neurologically intact state as a result of the operation and maintained preoperative ambulatory status. Moreover, in another report of a patient with C2 metastasis from thyroid cancer, posterior occipitocervical fixation resolved six months of chronic neck pain with a lack of recurrence or discomfort 11 months after surgery [9]. After surgery, radiation therapy was used in our patient for tumor control and significant pain palliation.

Achieving a bony fusion is challenging in the face of cancer and radiation treatment. The primary goals were palliation of pain and prevention of a neurological deficit (with realignment and stabilization) but not necessarily achieving long-term bony fusion, which is extremely challenging in the face of cancer and radiation. These goals were achieved. The patient did not suffer from any hardware failure prior to unfortunately expiring. Of note, we did not use human recombinant bone morphogenetic protein in this case since it is contraindicated for use in the vicinity of a resected or extant tumor or in patients with any active malignancy.

There are other modalities that may be used to palliate symptoms due to spinal metastases. Vertebroplasty has been used in patients with cervical metastases, although the metastases did not involve biomechanical instability [1,10,11]. Vertebroplasty is less invasive and is associated with rapid recovery, and a few cases have reported significant pain relief and improved quality of life for patients. Other techniques that have been used with success include aggressive transpedicular corpectomy, transoral approach, and lateral approach, but these approaches have been associated with higher rates of complication [6,11,12,19,20]. Overall, the use of a posterior approach is often more preferable to the upper cervical spine and cervical spine in general, providing adequate stabilization and relatively fewer complications compared to other approaches.

## Conclusions

We reported on a patient who presented with severe neck pain secondary to breast metastases causing a pathological fracture of the axis and atlantoaxial instability and subluxation. This was managed with preoperative reduction into appropriate alignment through crown halo traction and subsequent occipitocervical fusion and radiation therapy. This achieved long-term adequate palliation. This approach should be considered in patients who present with similar type pathological fractures of the axis that result in instability.
